# Feasibility and assessment of a comprehensive emergency department-based intervention for patients at risk of HIV

**DOI:** 10.1371/journal.pone.0310596

**Published:** 2024-09-26

**Authors:** Khaldia Osman, Joel Rodgers, Michael Fordham, Whitney Covington, Delissa T. Hand, Kelly Ross-Davis, Lauren A. Walter

**Affiliations:** 1 University of Alabama at Birmingham Heersink School of Medicine, Birmingham, Alabama, United States of America; 2 Division of Trauma and Acute Care Surgery, Department of Surgery, University of Alabama at Birmingham, Birmingham, AL, United States of America; 3 1917 (HIV) Clinic, University of Alabama at Birmingham, Birmingham, AL, United States of America; 4 Department of Emergency Medicine, University of Alabama at Birmingham, Birmingham, AL, United States of America; 5 O’Neal Comprehensive Cancer Center, University of Alabama at Birmingham, Birmingham, AL, United States of America; British Columbia Centre for Excellence in HIV/AIDS, CANADA

## Abstract

Behavioral factors increase the risk of contracting HIV. A comprehensive prevention services (CPS) intervention includes risk assessment and referral for those with confirmed risk. This project sought to assess the feasibility of an emergency department (ED)-based CPS program. A prospective cross-sectional assessment was conducted from October, 2021 through May, 2023, at a single ED in Birmingham, Alabama. Either of two screening methods were subjected to HIV negative adults: 1) manual chief complaint review or 2) objective electronic medical record (EMR) query. Manual and EMR screening methods considered sexually transmitted infections (STIs) or a positive urine drug test (to observe for commonly injectable drugs) within 12 months of current ED visit. Identified patients were approached in the ED (manual review) or via phone (EMR alert). Persons confirmed at risk for HIV following engagement questionnaire completion were made aware of their risk and offered referral to local CPS clinics. Primary outcome was CPS linkage. Descriptive analysis was performed. Of 184 patients approached, 147 agreed to engagement (79.9%), 117 in-person and 30 via phone; 125 (85.1%) were confirmed at risk for HIV; majority were white (66.4%), male (63.2%), between the ages of 30 and 49 (64.8%), uninsured (78.4%), and without a primary care provider (93.6%). Sexual behavior was identified as a recent (within six months) risk factor in 97 (77.6%) patients. Injection drug use was identified as a recent (within six months) risk factor in 71 (56.8%) patients. Fifty-four (43.2%) expressed interest in obtaining CPS follow-up. To-date, ten patients (18.5%) have connected with a CPS counsellor via phone and five (9.3%) have had a subsequent follow-up appointment to discuss CPS with a medical provider. Thirty at-risk patients (24.0%) received ED-initiated buprenorphine/naloxone. Targeted screening tools can aid in the identification of persons at risk for HIV in the ED; further, subsequent engagement and CPS implementation amongst this cohort is feasible. CPS clinic linkage may be challenging however, a CPS definition inclusive of ED-initiated medication for opioid use disorder, may offer opportunity for increased uptake.

## Introduction

HIV remains a persistent public health concern in the United States (US) [1]. While significant strides have been made in recent decades to End the Epidemic (EtE) in the United States (US), persistence of HIV suggests poor implementation of proven treatment advancements and highlights a gap in HIV prevention services [2]. Notably, in stark contrast to the rest of the country, the Southeast has not experienced a decrease in HIV incidence. While the South makes up less than 40% of the national population, it continues to account for over half of new HIV and AIDS cases [3]. In 2019, Alabama specifically ranked 10^th^ highest in HIV infection rates per 100,000 in the US, while only 24^th^ in rank by population [4, 5]. The significant prevalence of HIV in states categorized as the “Deep South” (including Alabama, Florida, Georgia, Louisiana, Mississippi, North Carolina, South Carolina, Tennessee, and Texas), is influenced to some extent by socioeconomic challenges such as poverty and unemployment; this region has the highest poverty rate and lowest median household income compared to other parts of the country. These economic disparities are linked to poorer health outcomes and potentially contribute to a greater incidence of HIV and other long-term illnesses [6].

Comprehensive HIV prevention services (CPS) have been identified as a key component to EtE [7]. A CPS intervention includes HIV behavioral risk assessment and subsequent prevention intervention options for those with confirmed risk factors, including unsafe sexual practices and/or injection drug use (IDU). A CPS intervention may include recommendation and referral for pre-exposure prophylaxis (PrEP). PrEP, when taken as prescribed, reduces the risk of getting HIV from sex by about 99% and by at least 74% among people who inject drugs [8]. Similar to previously reported regional disparities, the PrEP-to-Need Ratio (PnR) (calculated as the number of PrEP users divided by new HIV diagnoses) is lowest in the South, reflective of more unmet need [9]. More broadly, a CPS intervention for those confirmed at-risk for HIV could also include other pharmacologic options aimed directly at altering unsafe illicit drug use behavior; this would encompass buprenorphine/naloxone initiation in the setting of opioid use disorder (OUD) and IDU. First approved for OUD treatment in 2002, buprenorphine has been demonstrated to decrease illicit opioid use by 40% and improve long-term treatment retention in care by 75% [10].

The Emergency Department (ED) represents a unique medical setting open to all persons in the US and therefore, it often caters to cohorts who may not otherwise have access to traditional medical care or preventive health screening services; federal law ensures that even those without insurance cannot be refused emergency department treatment [11, 12]. With growing awareness of Emergency Medicine’s role in population health, an increasing number of EDs across the nation have engaged in universal HIV screening; further, a smaller number of EDs have implemented programs to assess patient HIV risk with subsequent definitive referral for those deemed PrEP-eligible [13, 14]. Separately, but concomitantly, amidst the opioid epidemic, EDs have become critical partners in the identification and management of OUD via OUD screening programs with subsequent ED-initiated medications for opioid use disorder (MOUD) (e.g., buprenorphine/naloxone) and referral to definitive addiction care [15].

Considering an expansive perspective of CPS, this project sought to assess the feasibility of an ED-based CPS program to identify and engage ED patients at-risk for HIV and offer broader, appropriate prevention referral and strategies. To our knowledge, this is the first assessment of its kind, which specifically considers a more expansive perspective of CPS, inclusive of but not limited to PrEP referral alone. Additionally, to the authors’ knowledge, at the time of composition, this represents the first reporting of an ED-based CPS program implemented in the Deep South.

## Methods

### Study setting

A prospective, cross-sectional assessment was conducted at the University of Alabama at Birmingham (UAB) ED from October, 2021 through May, 2023. UAB IRB approval was obtained for this project. The UAB Hospital is an academic, tertiary care medical center located in Birmingham, Alabama, an urban area with a metropolitan population exceeding 1.1 million. As part of UAB Hospital, the UAB ED provides care to Birmingham and the greater central Alabama region and receives 75,000 patient visits annually.

### Study design

The project definition for ‘at-risk for HIV’ was designed to align with nationally recognized HIV-risk variables including evidence of unsafe sexual behavior and/or evidence of illicit injection drug use [16]. Evidence of unsafe sexual behavior included diagnoses of sexually transmitted infections (STIs), specifically chlamydia, syphilis, gonorrhea, and trichomoniasis, either at the time of the documented ED visit or within 12 months before the visit.

Positive urine drug screens, either at the time of the documented ED visit or within 12 months prior, were utilized to assess risk of HIV acquisition through injection drug practices. Only illicit drugs commonly administered through injection (heroin, amphetamines, cocaine, and other opiates), as determined by the National Institute on Drug Abuse, were considered [17].

**Fig 1:**
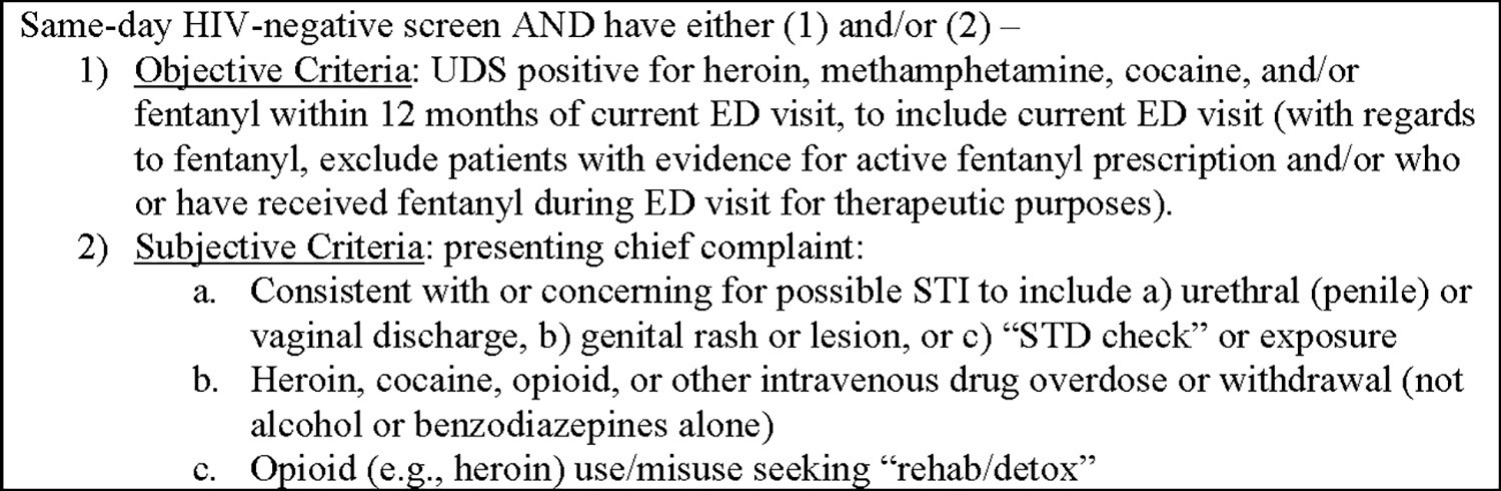
Manual, real-time chart review inclusion criteria.

The UAB ED offers non-targeted, opt-out HIV screening, for most adult patients (≥ 18 years-old to 75 years-old). Approximately one-quarter to one-third of the UAB ED population undergoes HIV screening. The most common algorithm reasons for non-screening include age parameter violation, known disease status and/or recent testing results available within the past 12 months. Persistent stigma associated with HIV also likely contributes to active patient opt-out [18]. For this project, all patients with negative HIV screens were then subjected to at least one of two potential screening methods to assess for HIV risk based upon identifiable behavioral risk factors. The methods utilized include: 1) manual, real-time chart review and 2) weekly, automated electronic medical record (EMR) reports. Considering the first method, when research assistants (RAs) were available, they monitored the ED tracking board to assess for inclusion and at risk criteria (Fig 1). To supplement variable and convenient RA monitoring, the second screening method included a weekly automated EMR at risk list which was generated from the same eligible ED cohort and considered objective laboratory data only, specifically urine drug screen (UDS) results and STI testing at the time of and within 12 months of ED visit.

Patients identified as potentially at risk by either RA real-time monitoring or via automated EMR query were eligible for RA engagement and assessment to confirm HIV risk. Patients identified real-time were approached in the ED; patients who were identified by EMR query, who had not previously been engaged in the ED, were eligible for HIV risk assessment via telephone (post-ED visit). Whether engaged in-person or via phone, RAs utilized a standardized script and questionnaire. Following RA introduction, verbal consent was obtained and documented in the study database prior to subsequent RA-participant engagement. A tablet interface was utilized in-person to deliver an ‘at risk for HIV’ questionnaire while an identical questionnaire was delivered verbally for phone contacts (S1 Addendum). Patients who indicated via the questionnaire that they had previously or were currently engaged in at risk behavior were asked to clarify the recency of the behavior (e.g., within the past week, within the past month, within the past three months, etc.). Patients with reported risk behavior within 12 months were considered to be confirmed at risk for HIV. Patients who were non-English speaking, deemed medically or psychiatrically unstable, or who were unable to provide verbal consent were excluded.

**Fig 2:**
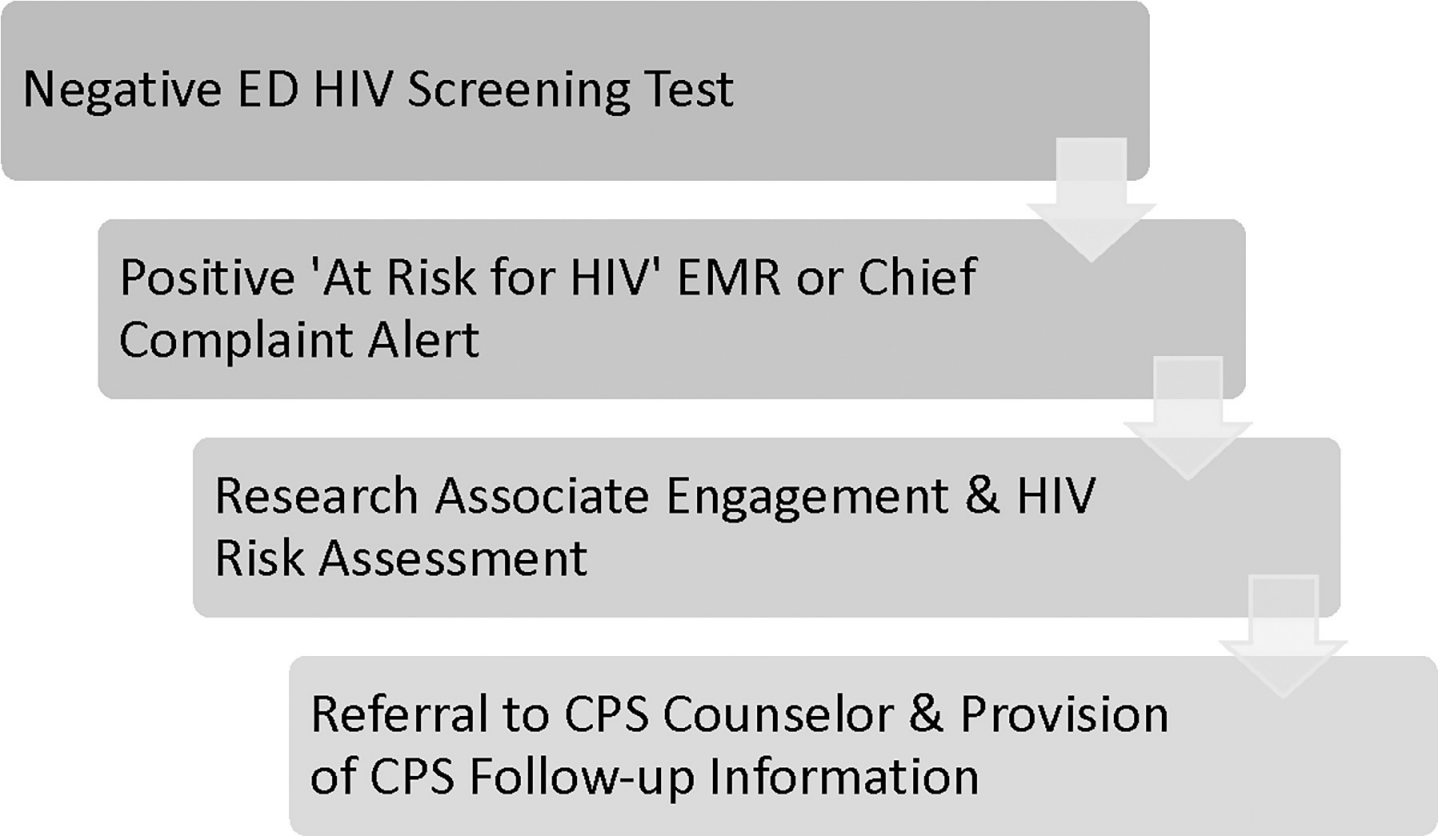
ED CPS project algorithm overview.

Participants confirmed at risk for HIV were made aware of their current HIV risk and queried about there awareness of PrEP specifically. At risk patients were offered direct connection to a PrEP/CPS counselor, facilitated real-time during PrEP clinic hours via phone; the PrEP/CPS counselor was notified via email and followed up post-ED visit via phone for those participants who expressed interest in counselor engagement after hours or on weekends (Fig 2). All patients who expressed interest were provided with referral information and handouts regarding local PrEP/CPS clinics [19].

### Data collection and study analyses

Descriptive patient demographics, to include age, gender, race/ethnicity, and insurance status, were collected. Patients with Medicaid, Medicare or both Medicaid and Medicare were identified as publicly insured. Prior establishment with a primary care provider (PCP) was also assessed. The primary outcome was participant uptake and categorization of CPS. Additional outcomes considered included confirmation and classification of risk status via utilized screening methods. PrEP initiation was not a captured data point or an included outcome measure.

All study data were collected and managed using REDCap, a secure, web-based data capture application [20]. All descriptive analyses were conducted using JMP Pro 16, SAS Institute Inc., Cary, NC, 1989–2023.

## Results

During the study time frame 19,985 ED HIV screening tests were conducted, 19,722 of which were negative. The total number of automated EMR query alerts received during the study period was 1,625, representing 1,625 patient visits. Overall, 184 unique ED patients were either approached in-person (67.9%) or via phone (32.1%) after at risk eligibility criterion was identified; those approached in-person were more likely to engage and complete subsequent confirmation of risk assessment. Amongst those flagged for engagement, injection or illicit drug use was the most common alert criteria identified, both subjectively via chief complaint (67.4%) as well as objectively via current (46.2%) or prior (23.9%) positive UDS (Table 1). Amongst confirmed at risk patients, over half reported IDU (52.0%) or unprotected intercourse (58.4%) within the last month (Table 2).

**Table 1:** ED CPS patient demographics & variables.

Patient	Approached/Called	Engaged	Confirmed Risk
Characteristic	n = 184	n = 147 (79.9%)	n = 125 (85.1%)
**Race:** *			
White	114 (62.0)	90 (61.2)	83 (66.4)
Black	65 (35.3)	52 (35.4)	38 (30.4)
Bi-/Multi-racial	3 (1.6)	3 (2.0)	2 (1.6)
**Ethnicity:** ^			
Non-Hispanic	177 (96.2)	140 (95.2)	118 (94.4)
Hispanic	4 (2.2)	4 (2.7)	4 (3.2)
**Gender:**			
Men	120 (65.2)	92 (62.6)	79 (63.2)
Women	64 (34.8)	55 (37.4)	46 (36.8)
**Age:**			
18–29	27 (14.7)	24 (16.3)	30 (24.0)
30–39	60 (32.6)	47 (32.0)	42 (33.6)
40–49	54 (29.3)	42 (28.6)	39 (31.2)
50–59	20 (10.9)	14 (9.5)	11 (8.8)
60–69	11 (6.0)	8 (5.4)	3 (2.4)
≥70	2 (1.1)	2 (1.4)	-
**Health Insurance:**			
Uninsured	149 (81.0)	126 (85.7)	98 (78.4)
Private/Commercial	13 (7.1)	8 (5.4)	6 (4.8)
Public	22 (12.0)	17 (11.6)	15 (12.0)
**Engagement Method**:			
In-Person (ED)	125 (67.9)	117 (79.6)	103 (82.4)
Via Phone	59 (32.1)	30 (20.4)	22 (17.6)
**Established PCP**	18 (9.8)	9 (6.1)	8 (6.4)
**Risk Flag**:			
Prior 12-mth +STI	17 (9.2)	14 (9.5)	13 (10.4)
This visit +STI	33 (17.9)	14 (9.5)	12 (9.6)
Prior 12-mth +UDS	44 (23.9)	37 (25.2)	37 (29.6)
This visit +UDS	85 (46.2)	57 (38.8)	47 (37.6)
Subjective STI CC	32 (17.4)	27 (14.7)	23 (18.4)
Subjective IDU CC	124 (67.4)	103 (70.1)	90 (72.0)

ED = emergency department; CPS = comprehensive prevention services; PCP = primary care provider; + = positive; STI = sexually transmitted infection; UDS = urine drug screen; CC = chief complaint

*two missing responses

^one missing responses

**Table 2:** HIV risk factor & recency amongst confirmed at risk, self-reported.

Risk Factor Recency	Total = 125
	n (%)
**Last Reported Unsafe/Unprotected Intercourse**	
Within the last week	48 (38.4)
Within the last month	25 (20.0)
Within the last 3 months	14 (11.2)
Within the last 6 months	10 (8.0)
Within the last 12 months	10 (8.0)
Greater than 12 months	11 (8.8)
Never	6 (4.8)
**Last Injection Drug Use**	
Within the last week	57 (45.6)
Within the last month	8 (6.4)
Within the last 3 months	2 (1.6)
Within the last 6 months	4 (3.2)
Within the last 12 months	5 (4.0)
Greater than 12 months	7 (5.6)
Never	42 (33.6)

Ninety-one (72.8%) confirmed at risk for HIV were aware of their potential risk for HIV acquisition; however, only 39 (31.2%) were aware of PrEP specifically. Fifty-four (43.2%) expressed interest in connecting with a medical professional to discuss CPS options. Ultimately, 10 (8.0%) confirmed at risk persons engaged with a PrEP counsellor, including seven during their ED visit and three via post-ED visit phone follow-up engagements. Five persons reported a post-ED in-person follow-up appointment with a medical professional to discuss PrEP/CPS specifically. These include three white, one Black, one Bi-/Multi-racial, two men and three women, one aged 18–29, one aged 30–39, and three aged 40–49; only one of these had health insurance (public). Four had sexual behavior as a risk factor; one had IDU risk behavior. No data is available to confirm subsequent PrEP initiation.

Thirty-nine of all approached (21.2%) received a buprenorphine/naloxone prescription from the ED, including 30 (24.0%) who were confirmed at risk for HIV. Considering specifically those confirmed at risk for HIV and with a chief complaint related to IDU, 27 (93.1%) were provided buprenorphine/naloxone at ED discharge.

## Discussion

This project demonstrates the capacity of an ED to accurately identify and engage with patients at risk for HIV. Considering specifically those who were willing to engage, the sensitivity of our combined screening methods was relatively high (85.0%), even when considering only the low-tech, chief complaint screening method (86.9%). The results of CPS uptake are somewhat dichotomous–while definitive PrEP/CPS clinic linkage by participants was low amongst all at risk (4.0% or 9.3% amongst those interested in CPS), when considering CPS more broadly, to include MOUD, particularly in an at risk cohort heavily impacted by IDU, uptake was more substantial. Hurdles to PrEP-specific clinic linkage and follow-up may be related to stigma associated with HIV, HIV risk behaviors or PrEP itself [21, 22]. Low PrEP clinic linkage may also be representative of significant social hurdles which impede capacity for and prioritization of PrEP clinic follow-up. The majority of those confirmed at risk (>90%) were either uninsured or publicly insured, a surrogate marker for social risk and social need [23]. Further, only 6.4% of those confirmed at risk had an established primary care provider (PCP). Persons with a PCP typically have regular access to traditional healthcare and often a trusted relationship with their healthcare provider; this scenario may foster open discussion regarding HIV risk and prevention options, including PrEP [24, 25]. Lack of a PCP may contribute to lack of risk awareness as well as lack of knowledge regarding available preventive measures, as was demonstrated in this cohort. Finally, it must also be recognized that this study took place amidst the COVID-19 pandemic, a time period during which both ED and HIV-related medical services were severely impacted [26]. This disruption extended to HIV CPS/PrEP services as well and was further amplified in the Deep South, the site of this project [27, 28]. Obstacles related to CPS/PrEP clinic capacity, patient engagement hesitation, and broader healthcare access issues dictated by the pandemic may have directly impacted definitive linkage success in this study [29, 30].

Low definitive linkage rates to CPS/PrEP clinic follow-up have been similarly demonstrated in prior ED-based PrEP referral studies, including a 2015–2017 study conducted at the University of Chicago (5.0% scheduled a PrEP clinic appointment and 2.2% initiated PrEP) and a 2018–2019 study conducted in a Baltimore ED (5.1% scheduled a PrEP clinic appointment and 1.3% initiated PrEP) [31, 32]. Removal of linkage steps, including placing a PrEP/CPS counsellor directly in the ED and/or empowering emergency physicians to write for/start PrEP directly, might be future considerations to increase CPS uptake. Barriers to these considerations include additional personnel expense as well as clinician time and buy-in.

Low definitive ED to PrEP linkage rates, reiterated in prior studies, has led to increased consideration and promotion of other CPS strategies, beyond PrEP, including consideration of MOUD, when appropriate. MOUD, classified as CPS given its potential to modify an individual’s IDU behavior, was initiated for nearly a quarter of those persons in this study found to be at risk for HIV and notably, the vast majority of those with IDU as an identified risk factor. This correlates with the high prevalence of IDU in this cohort specifically; IDU was almost four times more prevalent a risk flag than STI. A 2014 publication by Woody et. al, demonstrated that buprenorphine, “was a successful HIV risk reduction intervention for patients who remain in treatment, but with the added advantage of being accessible in settings other than methadone programs in the US.” [33]. Of note, this specific study’s publication pre-dates the declaration of the opioid epidemic as a public health crisis in the US (2017) [34]. Injection drug use currently accounts for 14% of cumulative HIV diagnoses in Alabama [35]. A recent report highlighting county-level vulnerability for rapid dissemination of HIV among persons who inject drugs (PWIDs) identified four Alabama counties in the top five percent of the US [36]. These unfortunate facts highlight the current impact of the opioid epidemic and IDU and demonstrate the link between Alabama’s opioid epidemic and HIV infection rates. EDs have been recognized as a major source of healthcare for people with OUD [37]. Opioid abusers are high resource utilizers of EDs, and PWID have been shown to utilize EDs over three times more frequently than the general population [38, 39]. This further highlights the ED as a strategic, potentially high-yield medical venue which exists at the overlap of OUD and HIV risk. Further, EDs often see patients who have limited or no access to other health services; for instance, only 1 in 16 confirmed at risk for HIV in this study had a PCP and greater than 75% were uninsured with likely limited access to a PCP. Finally, the more recent incorporation of ED-initiated efforts to address OUD directly, namely ED-initiated MOUD, may provide particular proximity for this form of CPS; this uptake may translate directly to future study designs considering ED-initiated or rapid start PrEP.

Clearly IDU is an overwhelming HIV risk factor in our cohort locally. Therefore, one might consider that programs which focus exclusively on IDU, to include ED-initiated OUD programs and/or safe injection use education, might be most efficacious in terms of HIV prevention impact. However, while there is some risk behavior overlap, this IDU-focused approach, in isolation, overlooks sexual risk behavior, and is in contrast with the prevalence of HIV acquisition risk factors seen in the US. Nationally, PWID represent a minority of new HIV cases annually while sexual exposure represents the vast majority [14]. Although STI flags were less frequent in this study, when questioned, over 75% of confirmed at risk reported unsafe sex within the past six months. Further, while this study’s reported follow-up numbers were small, three out of four who sought PrEP post-ED intervention had a documented sexual risk behavior in absence of IDU. A singular focus on IDU-based interventions, while clearly important, may miss a large at risk cohort. Given the prevalence of known national HIV acquisition risk factors, future study design may need to consider ways to increase capture of those persons with sexual risk behavior. Adoption of universal at risk ED HIV assessment, parallel to universal ED HIV screening, rather than a targeted intervention based upon screening flags, might improve capture of those with sexual risk; although, consideration of this approach does present additional time, personnel, and cost challenges.

While this project did not include a cost-effectiveness analysis, when considering the utility of an ED-directed CPS project beyond the research realm, sustainability and cost-benefit considerations are important from an institutional and public health perspective. ED-based HIV screening has already been previously demonstrated to be cost-effective [40, 41]. The addition of a CPS intervention to the algorithm for HIV negative screens would primarily include the cost of personnel, in the form of screeners/linkage coordinators and/or CPS counselors. Potential set-up costs to create an at risk flagging system, which could be variable based upon its level of complexity and intended breadth, might also exist. Subsequent costs to the patient, not necessarily incurred by the healthcare system, include the cost of medications (PrEP or MOUD) as well as the cost of follow-up visits with a PrEP clinic and/or for addiction care services. As noted above, the associated patient-specific costs may represent legitimate barriers to broader or more consistent uptake. Absorption of some of these costs by the healthcare system may improve uptake while still proving cost-effectiveness due to avoidance of HIV acquisition (and associated healthcare costs) [42, 43].

## Limitations

This is a single site study and therefore, the results may not be generalizable to all locations or institutions. The inclusion of a subjective chief complaint screening method, which was not able to be implemented around the clock, makes it challenging to define a specific total denominator for subjectively flagged patients, resulting in potential selection bias. Also, following up with patients post-ED visit, via phone, proved challenging. Many phone numbers were found to be not in service, erroneous, or resulted in multiple unanswered call attempts; this may also result in selection bias. Finally, UAB ED has engaged in an ED-initiated MOUD program since 2019. This parallel departmental practice, although recognized as a subspecialty standard of care and increasingly incorporated in EDs across the US, may result in higher numbers of MOUD provision than might be seen elsewhere, again, limiting generalizability [44]. Finally, as noted above in the discussion, the COVID-19 pandemic impacted both EDs and HIV services in the US on a broad scale. While our own ED and local CPS/PrEP referral clinics were operating at pre-pandemic capacity at the time this project launched (October, 2021), certainly patient-specific factors regarding willingness to engage and/or other persistent healthcare access issues influenced by the pandemic may have impacted follow-up and linkage.

## Conclusion

Relatively simple screening tools can aid in the accurate identification, engagement and referral of persons at risk for HIV in the ED. While definitive PrEP linkage was redemonstrated to be low in this Southern setting, a CPS intervention, particularly one that considers and incorporates MOUD for IDU, is feasible. Future work should strongly consider the potential linkage hurdles for the at risk cohort which likely include substantial social need. Longer term follow-up on the impact of an ED CPS intervention is needed to assess definitive impact. Again, while PrEP linkage numbers were low, CPS provision, in the form of MOUD, as well as the awareness and risk education supplied by the engagement itself, may have longer term implications for individual risk taking and therefore, HIV acquisition risk.

## Supporting information

S1 Addendum CPS RA engagement script. (DOCX)
