# Supplementary material for: Feasibility and assessment of a comprehensive emergency department-based intervention for patients at risk of HIV
Source: PLoS One. 2024 Sep 26;19(9):e0310596. doi: 10.1371/journal.pone.0310596 (PMC11426474; doi:10.1371/journal.pone.0310596)
Supplement: S1 Addendum — (DOCX) [file pone.0310596.s001.docx]

**S1 Addendum. CPS RA Engagement Script.**

"*Hi, I'm (insert name) and I use (insert pronouns) pronouns. I'm a research assistant with our emergency medicine research program here at UAB. As you may recall from triage, we screen all of our emergency patients for HIV; we also assess all of our patients for potential risk for HIV. Preventing HIV is one of the major ways we can help end the HIV epidemic. Your HIV screening test today was negative for HIV. Would it be okay if I asked you a few brief questions to see if you are at risk for HIV?*"

(If answer is “**yes**,” proceed with the following)

1. *When was the last time you used intravenous drugs?*

*When was the last time you had sex without a condom, including any times the condom broke, fell off, or was removed during sex?*

*Have you had chlamydia, gonorrhea, trichomoniasis, or syphilis in the past 12 months?*

Response(s) indicating intravenous drug use and/or unprotected sex within the past 12 months should prompt the following:

"*Are you currently on pre-exposure prophylaxis, or PrEP, for HIV*?

**If not**:

"1) *Are you aware of your current risk for HIV?*

*AND 2) Are you aware of PrEP, a medication which can reduce your chances of getting HIV?*

(For any response, script continues as follows:)

"*Would you be interested in connecting with a medical professional who can provide you with more information about how you can reduce your risk for HIV and even refer you for HIV preventive (PrEP) therapy*?"

An affirmative response would prompt engagement of 1917 PrEP clinic/coordinator, when available. If unavailable, please share the CDC PrEP handout, noting specifically the 1917 and Jefferson County Health Department PrEP clinic contact information listed on the back portion. In addition, in the event the PrEP clinic coordinator is unavailable, please confirm patient contact information and ask if it would be okay if they were contacted at a later time by a medical professional for additional risk counselling and to facilitate potential follow-up.

If they are not at high-risk based off of their responses to the three screening questions:

"*Based off of your responses, you are not currently at high risk for HIV, however, if you are still concerned about your potential risk or would be interested in more discussion about HIV risk factors and preventive measures, I would be happy to connect you to a medical professional with expertise in this area*."

(If requested, cue PrEP coordinator engagement, per above).

If engaging in-person, please offer all engaged patients the pocket CDC foldable pocket guide (with referral information).
